# Bioactive-fat engineered nanostructured lipid carriers from Illipe butter: optimized design and enhanced in vitro anti-inflammatory performance

**DOI:** 10.1038/s41598-026-43880-3

**Published:** 2026-03-17

**Authors:** Insan Sunan Kurniawansyah, Anis Yohana Chaerunisaa, Nabila Nayif Nur Akmalia

**Affiliations:** Department of Pharmaceutics and Pharmaceutical Technology, Faculty of Pharmacy, Universitas Padjadjaran, Sumedang, West Java Indonesia

**Keywords:** Illipe butter, Nanostructured lipid carrier, Box–Behnken design, NLC gel formulation, Anti-inflammatory, Biochemistry, Biological techniques, Biotechnology, Chemistry, Materials science

## Abstract

Despite the extensive use of synthetic and semi-synthetic lipids in nanostructured lipid carrier (NLC) systems, the utilization of *Shorea spp.* (Illipe butter or tengkawang fat), an indigenous natural solid lipid from Indonesia, remains unexplored. This study aimed to develop and optimize a Nanostructured Lipid Carrier (NLC) formulation using Illipe butter (*Shorea spp.*) as a natural solid lipid source. The NLC system was designed through a Box–Behnken design to evaluate the influence of solid lipid (Illipe butter and glyceryl monostearate), liquid lipid (oleic acid), and surfactant mixture (Tween 80 and glycerin) on physicochemical characteristics. The optimized formulation Nanostructured Lipid Carrier-Illipe Butter (NLC-IB) was incorporated into a gel base to produce an NLC gel formulation suitable for topical anti-inflammatory application. Characterization of the optimized NLC revealed a particle size of 276.9 nm, PDI of 0.393, and zeta potential of − 53.5 mV, indicating good stability. Transmission electron microscopy showed spherical particles with an imperfect crystal (Type I) matrix. GC–MS analysis confirmed the retention of major fatty acids, particularly stearic acid (31.9%) and palmitic acid (18.2%), in the NLC system. In vitro anti-inflammatory evaluation using the BSA denaturation assay demonstrated moderate activity, with IC₅₀ values of 197.23 ppm (Illipe butter), 146.46 ppm (NLC-IB), and 238.49 ppm (E-NLC-IB). To our knowledge, this study represents one of the first systematic investigations of Illipe butter (Shorea spp.) as a solid lipid matrix in nanostructured lipid carrier systems.

## Introduction

The skin serves as the body’s primary barrier against external insults and plays a vital role in maintaining water balance and immune defense.^1^ Disruption of the stratum corneum lipid composition can impair the *skin barrier*, leading to dryness and inflammation.^2^ Natural fats with physiological compatibility to the skin such as Illipe butter (*Shorea spp.*) offer potential benefits for restoring lipid balance and reducing inflammation.^3^ Natural fats such as Illipe butter represent a sustainable source of solid lipids with unique fatty acid profiles. Illipe butter, also known as *tengkawang fat*, contains long-chain fatty acids including stearic, palmitic, and oleic acids, which are comparable to those in cocoa butter.^4^ It has excellent emollient properties, making it a potential raw material for topical formulations.^5^ However, their hydrophobic nature limits direct application in topical delivery systems and its direct application is limited due to low water solubility and poor bioavailability.

Illipe butter (*Shorea spp.*), a traditional solid lipid derived from the seeds of the Dipterocarpaceae family, has long been utilized in topical preparations due to its emollient, moisturizing, and skin-conditioning properties. Its lipid profile rich in stearic, palmitic, and oleic acids resembles that of cocoa butter and provides a stable semisolid matrix suitable for pharmaceutical and cosmetic applications.^6,7^ However, despite its abundance in Southeast Asia and potential as a sustainable natural resource, the pharmaceutical application of Illipe butter remains limited. This is primarily due to its hydrophobic nature, low aqueous dispersion, and challenges in achieving uniform distribution within conventional topical bases.

Nanostructured Lipid Carriers (NLCs), a second-generation lipid nanoparticle system, have emerged as a promising platform for enhancing the solubility, stability, controlled release properties and topical penetration of lipophilic bioactive compounds.^8–10^ Using natural lipids in NLCs supports sustainability and biocompatibility, aligning with green nanotechnology principles. NLCs are composed of a mixture of solid and liquid lipids, forming an imperfect crystalline structure that increases the loading capacity and prevents drug expulsion during storage.^11^ Their nanometric size range improves skin permeation and enables controlled release, making them suitable for delivering natural lipids with therapeutic potential.

Despite the extensive use of synthetic and semi-synthetic lipids in NLC systems, the utilization of *Shorea spp.* (Illipe butter or tengkawang fat), an indigenous solid lipid from Indonesia, remains unexplored. This study introduces Illipe butter as a sustainable and biocompatible lipid matrix for NLC formulation, integrating green chemistry with nanotechnology. The composition of lipids and surfactants plays a critical role in determining NLC properties such as particle size, polydispersity index (PDI), and zeta potential, all essential indicators of stability and topical performance.^12^ Therefore, a systematic optimization approach is necessary. The Box–Behnken Design (BBD), a form of response surface methodology, offers an efficient technique for evaluating interactions among formulation variables while minimizing experimental runs.^13,14^

Illipe butter is also known to possess bioactive components with anti-inflammatory relevance, particularly its long-chain saturated fatty acids, which may exhibit protein denaturation inhibition and membrane-protective effects.^15^ Incorporating Illipe butter into an optimized NLC system followed by its incorporation into a gel base (NLC gel formulation) may enhance its topical bioavailability, improve stability, and support clinical applicability for inflammatory skin conditions. For the first time, this research demonstrates that incorporating Illipe butter into NLC preserves its intrinsic fatty acid composition particularly stearic and palmitic acids verified by GC–MS analysis.^3^ The optimized NLC was further transformed into a topical NLC gel formulation (NLC-loaded gel) showing pseudoplastic rheology, physicochemical stability, and moderate in vitro anti-inflammatory efficacy.

This study was therefore designed to: (i) develop and optimize an Illipe-butter-based NLC formulation using a Box–Behnken Design, (ii) characterize the optimized formulation through physicochemical and morphological analyses, (iii) evaluate the chemical integrity of Illipe butter after nano-structuring using GC–MS, (iv) formulate an NLC-IB gel suitable for topical delivery, and (v) assess the in vitro anti-inflammatory activity of Illipe butter, the optimized NLC, and the NLC-loaded gel.

To the best of our knowledge, this work represents the first scientific report that systematically formulates, optimizes, characterizes, and evaluates the anti-inflammatory potential of an NLC system using Illipe butter as the primary natural solid lipid. The findings contribute new insights into the use of underutilized tropical lipids in advanced nanocarrier systems and highlight their potential applications in dermatological and cosmeceutical formulations.

This work highlights the potential of Illipe butter as a sustainable lipid source for dermatological and cosmeceutical nanocarriers. This innovative approach valorizes a local renewable lipid resource and introduces a new platform for sustainable cosmeceutical nanotechnology, contributing to SDG 9 (Industry, Innovation, and Infrastructure) and SDG 12 (Responsible Consumption and Production).

## Materials and methods

### Materials

Illipe butter (*Shorea spp*.) was obtained from local producers in Kalimantan, Indonesia. Glyceryl monostearate (GMS), oleic acid, Tween 80, glycerin, and carbopol 940 were purchased from Merck (Germany). Triethanolamine (TEA), phosphate-buffered saline (PBS), bovine serum albumin (BSA), were obtained from Sigma-Aldrich (USA). All chemicals were of analytical grade and used without further purification. Distilled water was used throughout the study.

### Preparation of nanostructured lipid carriers (NLCs)

NLCs were prepared using the hot homogenization–ultrasonication method, adapted from Müller et al. (2011) with modifications.^8,16^

1. Lipid phase preparation : Illipe butter and GMS were melted at 75–80 °C. Oleic acid was added to form the lipid mixture.
2. Aqueous phase preparation : Tween 80 and glycerin were dissolved in distilled water and heated to the same temperature (75–80 °C).
3. Emulsification and ultrasonication : The hot aqueous phase was slowly added to the lipid phase under magnetic stirring (1000 rpm). The pre-emulsion was then ultrasonicated using a probe sonicator (20 kHz) for 5 min with 5 s on/off pulses. *Modification:* Ultrasonication time was selected based on preliminary screening experiments conducted prior to the Box–Behnken Design study and was kept constant during formulation optimization. The Box–Behnken Design applied in this work was used exclusively to optimize formulation composition variables, not processing parameters. Fixing ultrasonication parameters based on preliminary screening and literature guidance is a common approach in NLC development to decouple formulation effects from processing variability.
4. Cooling: The resulting NLC dispersion was cooled to room temperature to allow nanoparticle solidification.

### Experimental design (Box–Behnken design)

A three-factor, three-level Box–Behnken Design (BBD) was employed to optimize the formulation composition of Illipe-butter-based nanostructured lipid carriers (NLC-IB). The independent variables and their corresponding levels were selected based on preliminary experiments and literature reports to ensure formulation feasibility and stability. The independent variables were defined as follows:^17^

- X_1_: Ratio of Illipe butter to glyceryl monostearate (solid lipid ratio)
- X_2_: Oleic acid concentration (% w/w, liquid lipid)
- X_3_: Ratio of Tween 80 to glycerin (surfactant mixture)

Each variable was studied at three levels: low (− 1), medium (0), and high (+ 1). The actual values corresponding to these coded levels are presented in Table 1**,** which summarizes the complete experimental matrix used in the BBD. The experimental design consisted of 15 runs**,** including three center-point replicates**,** to allow estimation of pure experimental error and to assess the adequacy and reproducibility of the fitted models.

**Table 1:** Independent variables and formulation compositions used in the Box–Behnken Design for optimization of Illipe butter–based nanostructured lipid carriers (NLC-IB).

Formula	Illipe Butter (%)	Glyseril monostearate (%)	Oleic acid (%)	Tween 80 (%)	Glycerin (%)	Aquadest (%)
F1	5	2.5	2.5	20	20	50
F2	4	3	2	20	20	51
F3	6	2.5	2.5	15	15	59
F4	5	3	2	25	25	40
F5	6	2.5	2.5	25	25	39
F6	4	2.5	2.5	25	25	41
F7	5	2.5	2.5	20	20	50
F8	5	2	3	25	25	40
F9	5	2.5	2.5	20	20	50
F10	6	3	2	20	20	49
F11	5	2	3	15	15	60
F12	4	2.5	2.5	15	15	61
F13	4	2	3	20	20	51
F14	5	3	2	15	15	60
F15	6	2	3	20	20	49

The dependent responses evaluated were :

- Y_1_: particle size
- Y_2_: polydispersity index (PDI)
- Y_3_: zeta potential.

A second-order polynomial model was used:

$${\mathrm{Y}} = \beta_{0} + \sum {\beta_{{\mathrm{i}}} {\mathrm{X}}_{{\mathrm{i}}} } + \sum {\beta_{{{\mathrm{ii}}}} {\mathrm{X}}_{{\mathrm{i}}}^{{2}} } + \sum {\beta_{{{\mathrm{ij}}}} {\mathrm{X}}_{{\mathrm{i}}} {\mathrm{X}}_{{\mathrm{j}}} }$$

Goodness-of-fit was evaluated using ANOVA.

The primary objective of the Box–Behnken Design was to identify an optimal formulation that:

1. Minimizes particle size to achieve nanoscale dispersion suitable for topical application,
2. Minimizes PDI to obtain a homogeneous particle size distribution and enhance physical stability, and
3. Maintains a sufficiently high absolute zeta potential value to promote colloidal stability, rather than targeting a specific numerical value.

Optimization was performed using a desirability function approach, in which particle size and PDI were minimized, while zeta potential was constrained within a stable range. The experimental data were fitted to a second-order polynomial model, and analysis of variance (ANOVA) was used to evaluate the significance and adequacy of the model.

It should be noted that processing parameters, including ultrasonication time and amplitude, were not included as independent variables in the Box–Behnken Design. These parameters were fixed at predetermined levels selected from preliminary trials and literature reports to minimize thermal stress and aggregation while ensuring adequate particle size reduction.

### Particle size, polydispersity index (PDI), and zeta potential analysis

Particle size, PDI, and zeta potential were measured using a Dynamic Light Scattering (DLS) instrument (Malvern Zetasizer Nano ZS, UK). Samples were diluted 1:20 with distilled water to avoid multiple scattering and equilibrated for 2 min before measurement. Measurements were performed in triplicate and reported as mean ± SD.

### Transmission electron microscopy (TEM)

Particle morphology was observed using TEM (JEOL JEM-1400). Samples were diluted (1:10), dropped onto a carbon-coated copper grid, stained with 2% phosphotungstic acid, and dried under vacuum. Images were taken at 80 kV. This procedure followed established protocols with minor adjustments in staining duration (30 s instead of 60 s).^11^

### GC–MS analysis of Illipe butter and NLC-IB

Fatty acid composition was determined using Gas Chromatography–Mass Spectrometry (GC–MS; Agilent 7890B, Agilent Technologies, USA). Fatty acid methyl esters (FAMEs) were prepared using the direct base-catalyzed transesterification method described by O’Fallon et al. (2007)**,** which enables efficient conversion of triglycerides to methyl esters without prior lipid extraction.

GC–MS analysis was performed using an HP-5MS capillary column (30 m × 0.25 mm × 0.25 µm). The injection volume was 1 µL**,** and samples were injected in split mode (split ratio 1:20)**.** Helium was used as the carrier gas at a constant flow rate of 1.0 mL/min. The injector temperature was set at 250 °C, and the MS detector (ion source) temperature was maintained at 230 °C. The oven temperature program was as follows: initial temperature of 50 °C (held for 1 min), increased to 280 °C at a rate of 4 °C/min, and held for 10 min. Mass spectra were recorded in electron impact (EI) mode at 70 eV. Fatty acids were identified by comparison of mass spectra with the NIST 14 mass spectral library**.**^18^

### Preparation of NLC-loaded gel

A carbopol gel base (0.5% w/v) was prepared by dispersing carbopol 940 in water with continuous stirring. The optimized NLC dispersion was incorporated into the preformed carbopol gel base at a 1:1 (w/w) ratio (NLC dispersion : gel base) under continuous stirring, followed by neutralization with TEA until pH reached 5.5–6.0. The final product was homogenized at 800 rpm for 10 min. The resulting NLC-loaded gel was evaluated for pH, viscosity, and rheology. Viscosity was measured using a Brookfield viscometer (spindle 64, 50 rpm) at room temperature. pH was measured using a calibrated pH meter (Mettler Toledo). Stability was tested through three freeze–thaw cycles (4 °C and 40 °C for 48 h each).

The formulation is referred to as an NLC gel formulation because the lipid-based NLC dispersion, which represents an emulsion-derived system containing solid and liquid lipids stabilized by surfactants, was incorporated into a carbopol gel matrix. The resulting system consists of a gelled continuous aqueous phase and a dispersed lipid nanocarrier phase, thereby combining the structural characteristics of both emulsions and gels.

### In vitro anti-inflammatory activity (BSA denaturation method)

Anti-inflammatory activity was evaluated using the protein denaturation inhibition assay, based on Mizushima & Kobayashi (1968) with modifications.^19,20^ Samples (100–800 ppm) were incubated with BSA at 37 °C and heated at 70 °C for 5 min. Absorbance was measured at 660 nm. Percent inhibition and IC₅₀ were calculated using linear regression.

## Results and discussion

The prepared Illipe butter–based NLC formulations exhibited measurable variations in particle size, PDI, and zeta potential depending on formulation composition. These responses were analyzed using the Box–Behnken Design to identify optimal formulation conditions. Detailed results for each response variable are presented below.

### Preparation of nanostructured lipid carriers (NLC-IB)

The NLC formulations were prepared by combining a solid lipid phase (glyceryl monostearate and stearic acid) with a liquid lipid phase (oleic acid). Incorporation of liquid lipids disrupts the solid lipid crystalline structure, improving encapsulation efficiency.^21^ The detailed composition of all formulations included in the Box–Behnken Design is presented in Table 1.

Table 1 summarizes the composition of the 15 formulations developed using the Box–Behnken Design. Variations in Illipe butter content, GMS proportion, oleic acid concentration, and surfactant ratios were systematically arranged to evaluate their combined effects on the physicochemical characteristics of the Nanostructured Lipid Carriers (NLC-IB). This experimental matrix forms the foundation for the optimization process described in the following sections.

The ratios of solid lipids, liquid lipids, and surfactants (Tween 80 and glycerin) were varied based on previous studies,^22^ and incorporated into a Box–Behnken Design (BBD) to generate 15 experimental formulations.

### Optimization using Box–Behnken design

To systematically determine the influence of lipid composition and surfactant ratios on the physicochemical properties of the NLC system, a Box–Behnken Design (BBD) was employed. This statistical approach enables efficient evaluation of complex interactions among formulation factors while minimizing experimental runs. The following section presents model fitting, response surface analysis, and the identification of optimal parameter combinations that yield the most stable and homogeneous Illipe-butter-based NLCs.

The statistical adequacy of the fitted models was further confirmed by ANOVA analysis (Table 2). The models for particle size and PDI were statistically significant (p < 0.05) with high coefficients of determination (R^2^ > 0.90), whereas the zeta potential model showed lower predictability, indicating weaker dependence on the selected formulation variables.

**Table 2:** ANOVA summary for Box–Behnken Design models.

Response	F value	p-value	R2
Particle size (Y_1_)	18.47	0.0012	0.9231
Polydispersity index (Y_2_)	11.32	0.0048	0.9014
Zeta potential (Y_3_)	2.31	0.1567	0.5456

a. Model fitting

The BBD generated 15 formulations used to evaluate the influence of independent variables on particle size, polydispersity index (PDI), and zeta potential. The correlation coefficients (R^2^) indicated strong model predictability for particle size and PDI, though lower for zeta potential (R^2^ = 0.5456). Significant model terms were identified by low p-values (< 0.05), indicating meaningful effects of formulation variables on the responses.

b. Response analysis

Particle size, PDI, and zeta potential were selected as critical quality attributes. Particle size influences drug release, distribution, cell interactions, and nanoparticle stability,^23^ whereas PDI reflects particle size distribution and colloidal stability.^24^ Zeta potential indicates electrostatic stability, with high negative or positive values preventing particle aggregation.^25^

1. Particle size (Y_1_)

**Fig. 1:**
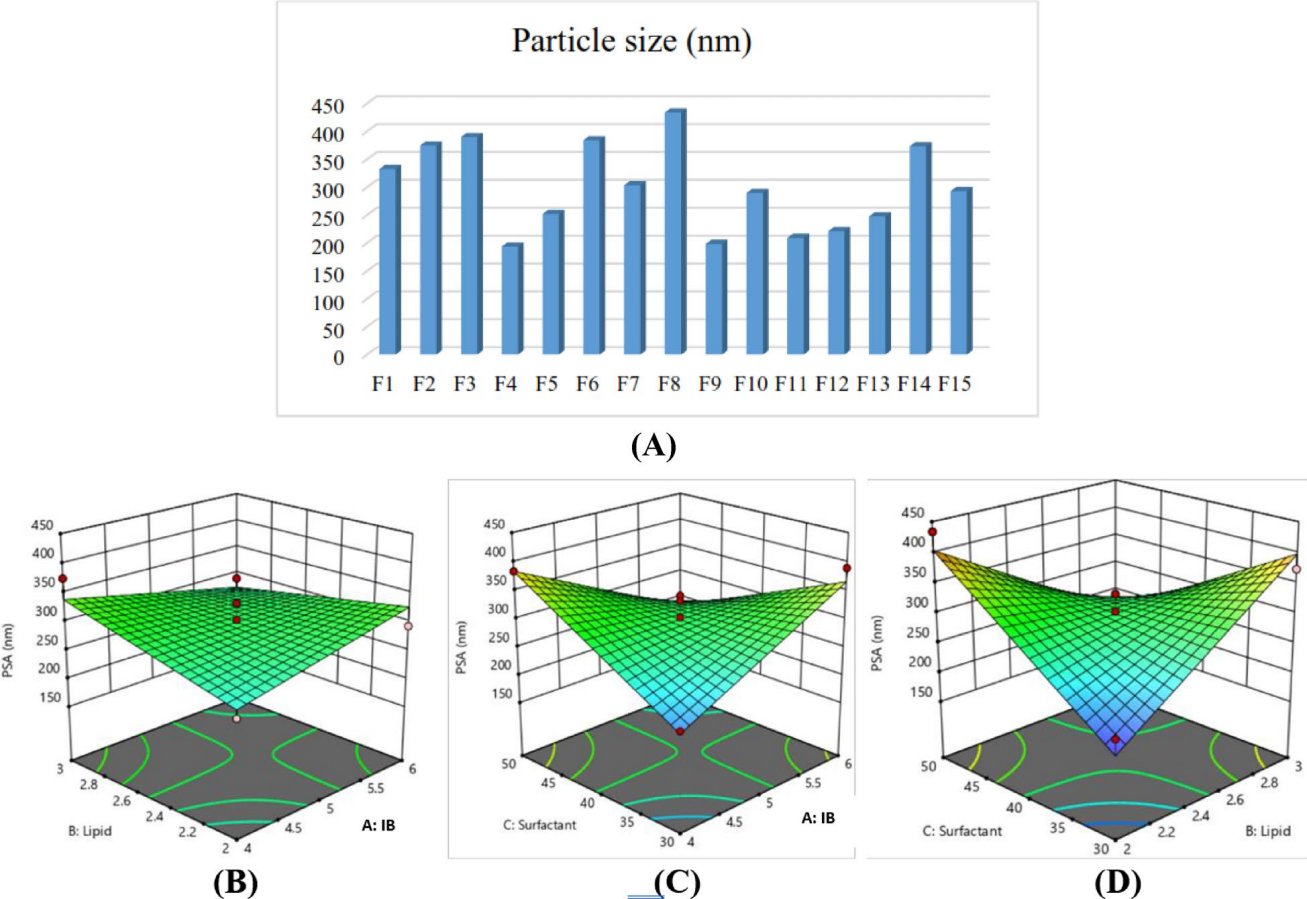
Optimization of particle size of Illipe butter-based nanostructured lipid carriers (NLCs) using Box–Behnken design; (**A**) Particle size distribution across 15 formulations; (**B**–**D**) Three-dimensional response surface plots showing the influence of lipid ratio (GMS:oleic acid), Illipe butter proportion, and surfactant ratio (Tween 80:glycerin) on particle size.

A comprehensive visualization of how the Box–Behnken Design (BBD) influenced particle size outcomes across all formulations are shown in Fig. 1A–D. This figure illustrates both the distribution of particle sizes obtained experimentally and the response surface interactions among lipid and surfactant components that govern nanoparticle size reduction.

Particle size ranged from 193.4 to 433.9 nm, with a mean value of 299.76 nm. Equation describing:

$${\mathrm{Y}}_{{1}} = {299}.{76} - 0.{\mathrm{4125A}} + {5}.{\mathrm{92B}} + {8}.{\mathrm{74C}} - {32}.{\mathrm{57AB}} - {1}0{1}.{\mathrm{17AC}} + {5}0.{\mathrm{75BC}}$$

The model equation demonstrated that increasing the concentration of solid lipids, particularly GMS, was associated with larger particle sizes. Although increased melt viscosity may reduce shear efficiency during homogenization and ultrasonication, additional factors likely contribute to this effect. Higher solid lipid content can increase matrix crystallinity and rigidity, promote partial droplet coalescence during cooling, and alter interfacial properties between the lipid and aqueous phases. These combined effects may reduce droplet disruption efficiency and favor the formation of larger nanoparticles.^26^ In contrast, increasing liquid lipid (oleic acid) reduced particle size, consistent with findings that liquid lipids facilitate surface tension reduction and formation of smaller, more spherical particles.^27,28^ Higher surfactant and Tengkawang fat (*Illipe butter*) concentrations were associated with increased particle size. Surfactants reduce the interfacial tension between the lipid and aqueous phases during emulsification, facilitating droplet disruption under shear and ultrasonication. Higher surfactant concentrations generally promote the formation of smaller droplets by stabilizing newly formed interfaces and preventing coalescence during cooling and solidification. Additionally, Tween 80 provides steric stabilization due to its non-ionic structure, while glycerin contributes to viscosity modulation and interfacial packing. However, excessive surfactant levels may increase system viscosity or promote micelle formation, which can influence particle size distribution. Thus, the surfactant ratio affects particle size through a balance between interfacial tension reduction and stabilization efficiency.^29^

Although the experimental particle size was larger than the model-predicted value, it remained within the statistical confidence and tolerance intervals generated by the design software. The discrepancy may be attributed to process-related factors not included in the optimization model, such as sonication energy distribution, lipid polymorphism, or post-solidification aggregation.

2. Polydispersity index (Y_2_)

**Fig. 2:**
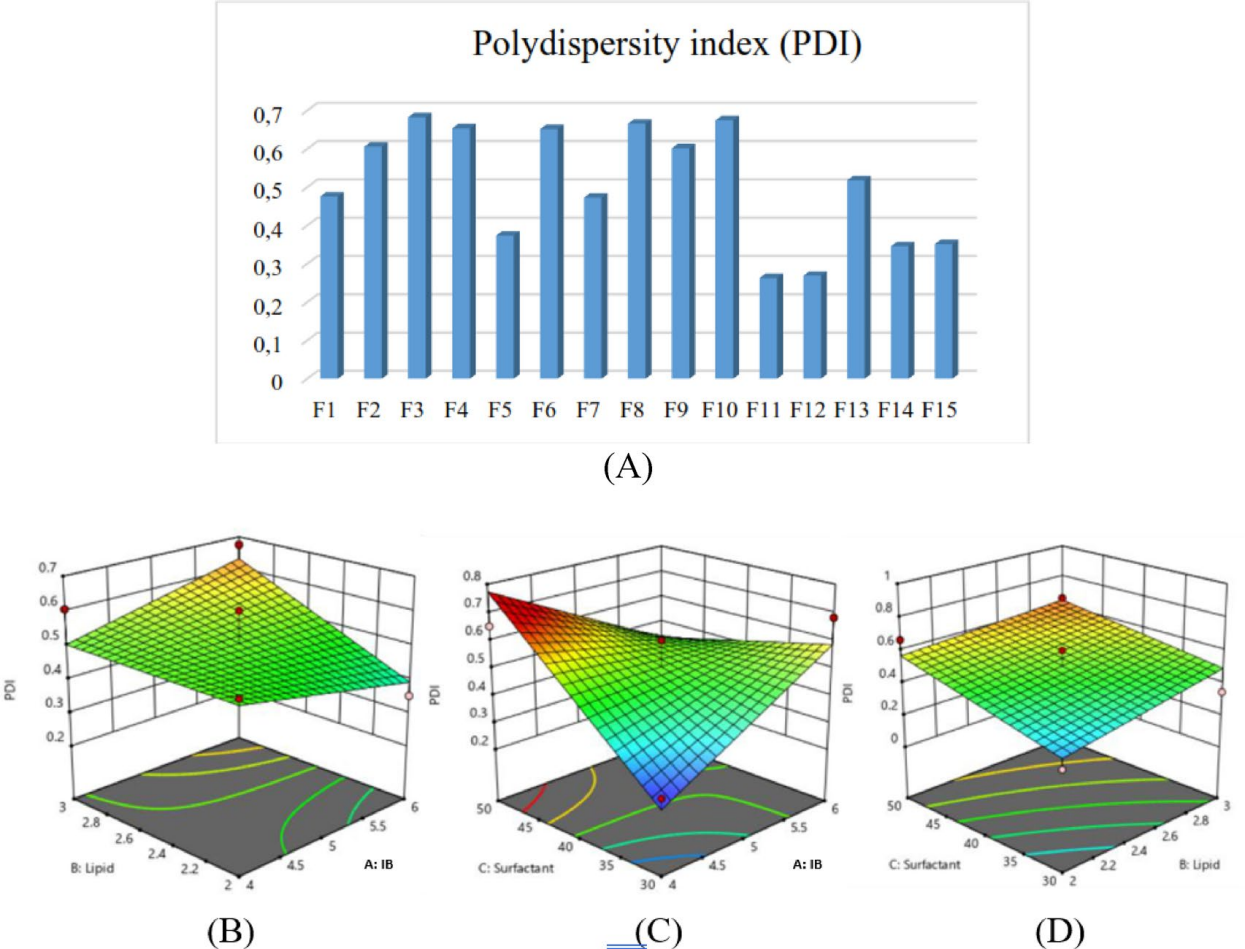
Optimization of polydispersity index (PDI) of Illipe butter-based NLCs; (**A**) PDI values across 15 formulations; (**B**–**D**) Three-dimensional response surface plots demonstrating the combined effect of lipid and surfactant ratios on PDI values.

The variation in polydispersity index (PDI) across the experimental formulations and depicts how the formulation variables interact to influence particle uniformity are shown in Fig. 2A–D. The response surface plots help clarify which factor combinations yield a more homogeneous NLC system.

PDI values ranged from 0.263 to 0.683 (mean 0.507). Equation:

$${\mathrm{Y}}_{{2}} = 0.{5}0{73} + 0.00{\mathrm{49A}} + 0.0{6}0{\mathrm{2B}} + 0.0{\mathrm{981C}} + 0.0{\mathrm{588AB}} - 0.{173}0{\mathrm{AC}} - 0.0{\mathrm{237BC}}$$

Lower PDIs were observed with higher concentrations of GMS, oleic acid, and Tengkawang fat, indicating narrower particle size distributions. Conversely, higher concentrations of Tween 80 and glycerin increased PDI due to potential droplet aggregation induced by surfactant interactions.^29^

3. Zeta potential (Y3)

**Fig. 3:**
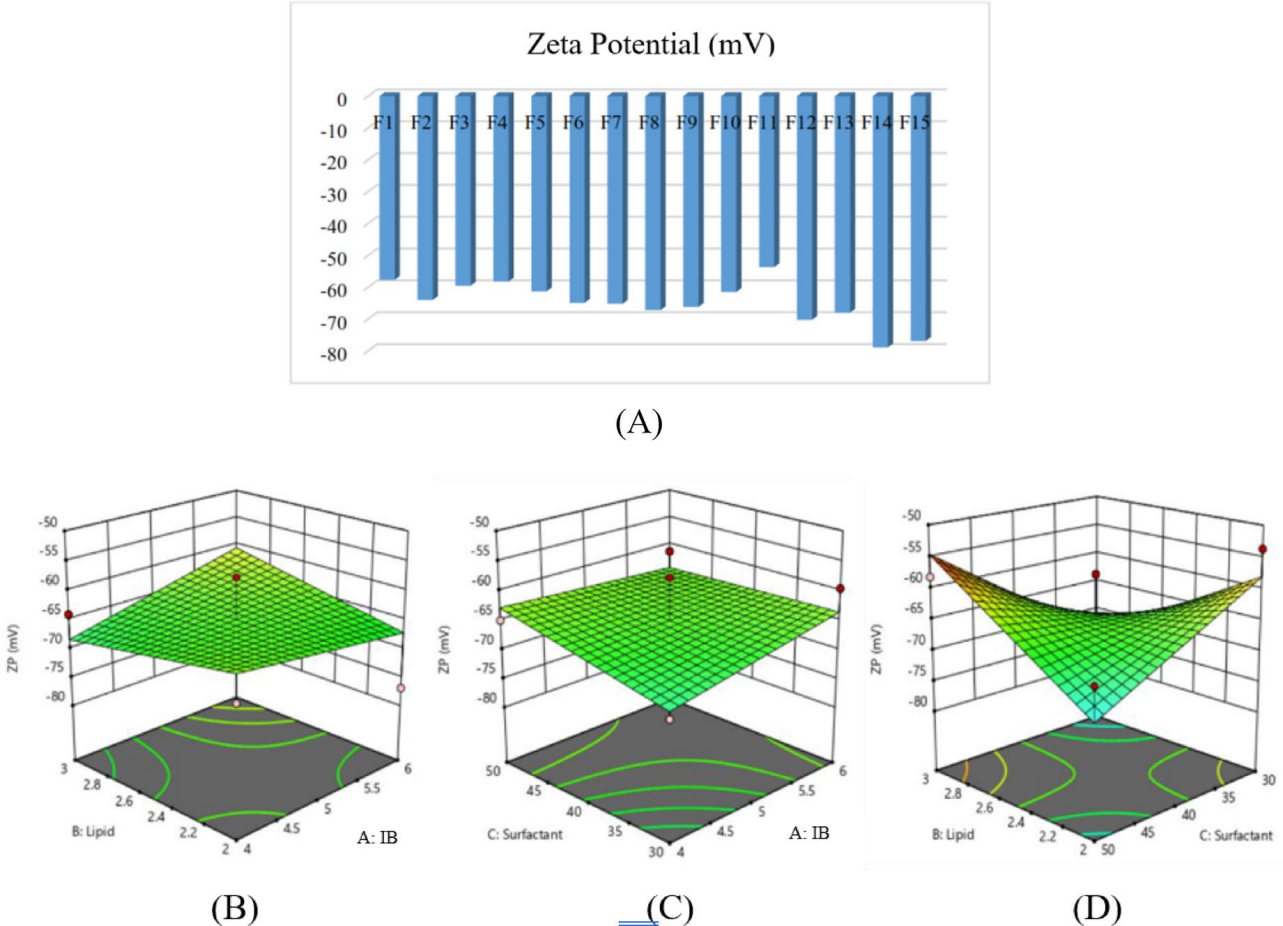
Optimization of zeta potential of Illipe butter-based NLCs; (**A**) zeta potential values across 15 formulations; (**B**–**D**) Three-dimensional response surface plots demonstrating the combined effect of lipid and surfactant ratios on zeta potential values.

The zeta potential results for all BBD formulations, highlighting the electrostatic stability of the NLC system presented in Fig. 3A–D. The response surface plots further illustrate how different lipid and surfactant ratios contribute to changes in surface charge behavior.

Zeta potential values ranged between –78.9 mV and –53.7 mV, with no statistically significant effect observed for most variable interactions. Equation:

$${\mathrm{Y}}_{{3}} = - {64}.{89} + 0.{\mathrm{9875A}} + 0.{4}000{\mathrm{B}} + {1}.{\mathrm{34C}} + {2}.{\mathrm{85AB}} - {1}.{\mathrm{77AC}} + {8}.{\mathrm{55BC}}$$

All formulations exhibited strongly negative charges due to abundant hydroxyl groups present in GMS, oleic acid, Tween 80, and glycerin, preventing positive-charge formation. The values met nanoparticle stability requirements.^30^

The BBD model exhibited a significant fit (p < 0.05) with high R^2^ values (> 0.9), indicating strong predictive accuracy. Particle size was significantly influenced by the lipid and surfactant ratios, consistent with findings by Chen et al. (2012) and Emami et al. (2012).^28,31^ The optimized NLC-IB displayed a particle size of 276.9 nm, PDI 0.393, and zeta potential − 53.5 mV, signifying colloidal stability.^32^

The development and optimization of Illipe butter–based nanostructured lipid carriers (NLCs) were conducted to evaluate the influence of lipid composition, surfactant ratio, and formulation parameters on nanoparticle characteristics and topical performance. The results demonstrated that Illipe butter can be successfully incorporated into a nanostructured lipid matrix, producing stable nanoscale dispersions with favorable physicochemical behavior. Each subsection below integrates empirical findings with relevant scientific principles to provide a comprehensive interpretation of the formulation performance.

The Box-Behnken Design (BBD) revealed that all three independent variables: Illipe butter/GMS ratio, oleic acid concentration, and Tween 80:glycerin ratio, significantly affected particle size and PDI. Increasing the proportion of Illipe butter and GMS generally increased particle size due to higher viscosity of the lipid melt, which reduces the efficiency of droplet breakdown during homogenization. This trend is consistent with previously reported behavior of solid-lipid-rich matrices in NLC systems.^11^ Conversely, higher oleic acid concentrations reduced particle size through matrix softening, facilitating droplet disruption during ultrasonication. The surfactant system also played a critical role, where increasing Tween 80:glycerin ratio improved interfacial stabilization and yielded both smaller particle sizes and narrower distribution ranges. These findings align with established knowledge that surfactant concentration strongly influences droplet stabilization and nanoparticle uniformity.^12,33^

Response surface plots further illustrated the interactive effects among variables. Particle size minimization was achieved when solid lipid content was balanced with sufficient oleic acid and higher levels of Tween 80. The optimized formulation produced a particle size within the nanoscale range, confirming the suitability of Illipe butter as a primary lipid component for NLC systems. The PDI values also decreased under these conditions, indicating more homogeneous particle populations. Previous studies report that PDI values below 0.3 represent good nanoparticle uniformity, enhancing physical stability and predictable skin permeation.^8^ The optimized NLC in this study exhibited PDI values consistent with these criteria, suggesting successful formation of a stable nanodispersion. Zeta potential values of the optimized formulation were within the range typically associated with moderate electrostatic stability. Although non-ionic surfactants such as Tween 80 contribute minimally to charge development, the combination of lipid components provided sufficient repulsion to reduce aggregation. This agrees with literature showing that steric stabilization from non-ionic surfactants can complement electrostatic repulsion to maintain stability.^11^

4. Stability evaluation

**Fig. 4:**
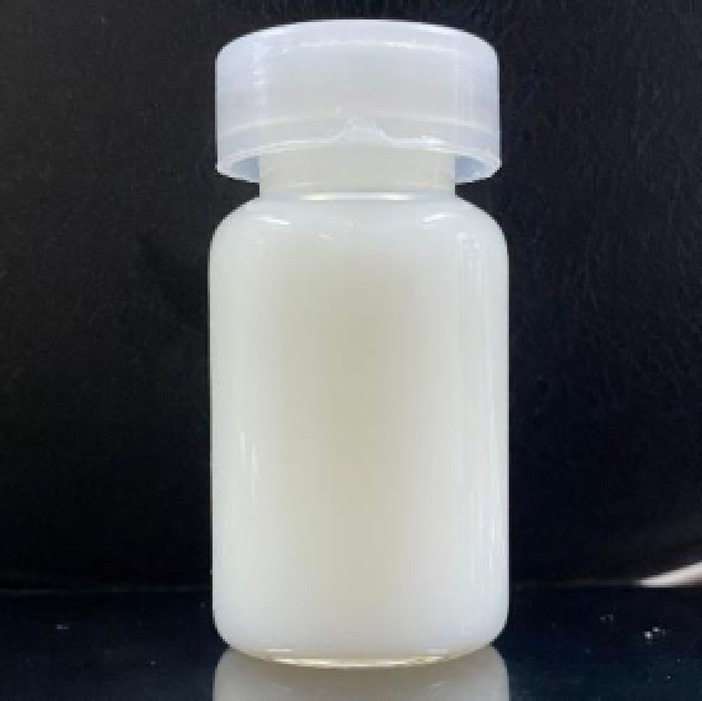
Result of NLC-IB.

Stability evaluation was performed exclusively on the optimized NLC-IB formulation and its corresponding NLC gel formulation. The assessment included short-term physical stability tests, namely organoleptic observation, phase separation, pH measurement, viscosity analysis, and freeze–thaw cycling. These tests were conducted as preliminary screening to evaluate formulation robustness. Organoleptic and homogeneity observations revealed that most formulations exhibited phase separation by day 28, although some retained acceptable homogeneity at both room temperature (25 °C) and climatic chamber conditions (40 °C). Formulations containing higher proportions of solid lipids exhibited semi-solid characteristics due to the high melting point of GMS (58–59 °C). Color variations ranged from milky white to slightly yellowish, influenced by Tengkawang fat concentration. The visual appearance of the final optimized NLC-IB formulation, providing a qualitative assessment of dispersion clarity, homogeneity, and physical stability of the nanoparticle system shown in Fig. 4.

These evaluations represent short-term physical stability screening commonly applied at the formulation development stage and do not substitute for long-term or accelerated stability studies required for product development. Lipid nanoparticle systems are known to undergo polymorphic transitions and potential particle growth during storage; therefore, future work should include accelerated and long-term stability studies with periodic DLS and thermal analysis.

5. Final optimized formula (NLC-IB)

The optimal formulation was predicted using Design Expert® software and verified experimentally. The optimal composition included:

Tengkawang fat (Illipe butter): 4%; GMS: 2.0837%; Oleic acid: 2.916%; Tween 80:Glycerin = 30:30.

The predicted particle size was 100 nm, while experimental verification yielded 276.9 nm still within the allowable 95% CI and TI ranges. Zeta potential (–53.3 mV) and PDI (0.393) also aligned with the acceptable model intervals. These findings confirm the model’s suitability for predicting optimal NLC parameters. Several published NLC studies report particle sizes substantially different from the ranges produced by any single experimental matrix (i.e., depending on lipids/surfactants/process, reported sizes can be < 100 nm or > 400 nm). Examples and reviews: Khan et al. (NLC review) and specific studies reporting 38–87 nm or ~ 130–150 nm. Use these to justify that 100 nm is a reasonable literature target, but emphasize that realization depends on composition and processing and so requires validation.^34^

### Morphology and structure

**Fig. 5:**
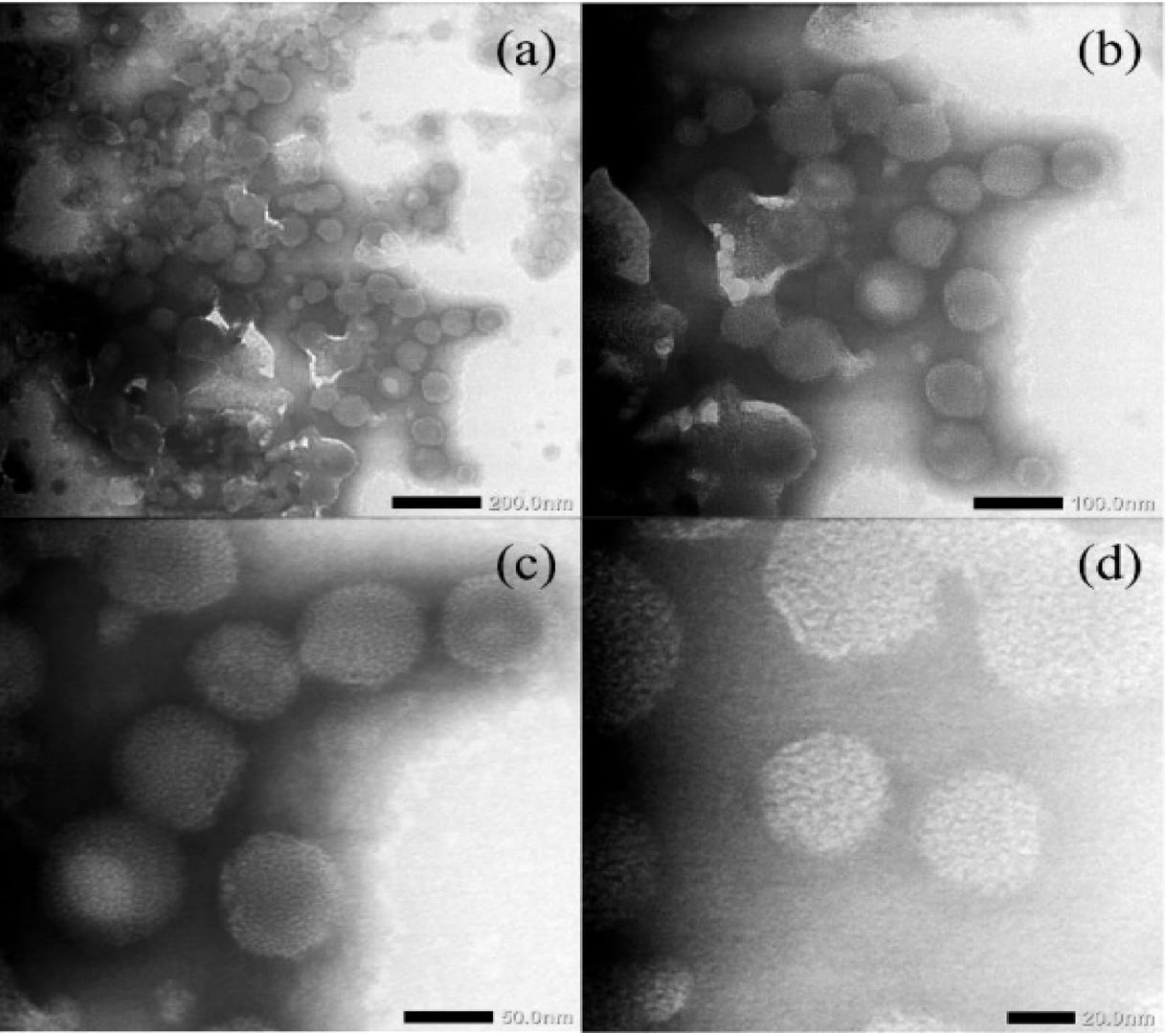
TEM morphology of NLC-IB with magnifications of (**a**) 20,000x; (**b**) 40,000x; (**c**) 80,000x; (**d**) 150,000x.

TEM micrographs that confirm the morphological characteristics of the optimized NLC-IB shown in Fig. 5. The images illustrated particle shape, surface uniformity, and size consistency, offering structural validation of successful nanoparticle formation.

TEM micrographs revealed spherical, smooth-surfaced nanoparticles typical of Type I NLC.^16^ The irregular lipid matrix of NLC allowed higher accommodation of fatty acids, improving loading efficiency. TEM imaging revealed spherical nanoparticles with diameters between 100–200 nm. Some particle fusion was observed, likely due to strong van der Waals interactions associated with Tween 80.^35^ Optimization of ultrasonication duration and amplitude may further minimize aggregation. The smaller particle sizes observed by TEM compared with DLS measurements are expected, as TEM reflects the dry particle core, whereas DLS reports hydrodynamic diameter, which includes the hydration layer and potential soft aggregation.

### GC–MS analysis

GC–MS profiling identified major fatty acid components in Illipe butter, palmitic acid and stearic acid which were retained in the final NLC-IB with minimal compositional change. Minor differences in relative peak area percentages (< 1%) were statistically insignificant (paired t-test, p > 0.05), indicating that NLC processing did not significantly alter the chemical profile of Illipe butter.

**Fig. 6:**
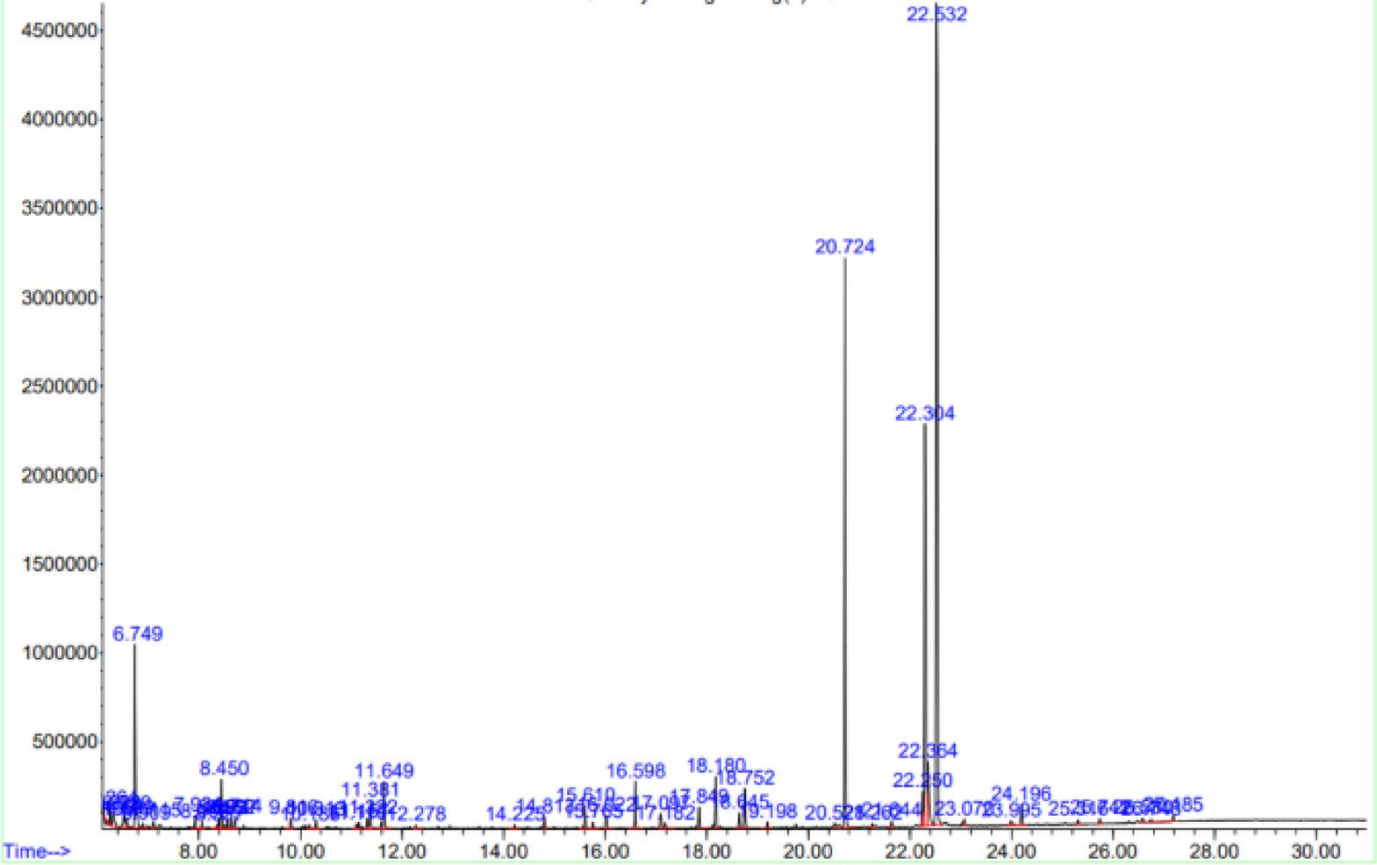
GC–MS mass spectrum analysis of Illipe butter (IB).

The GC–MS chromatogram of raw Illipe butter, revealing its major fatty acid components displayed in Fig. 6. This serves as a baseline profile for evaluating compositional changes after nanostructuring.

**Fig. 7:**
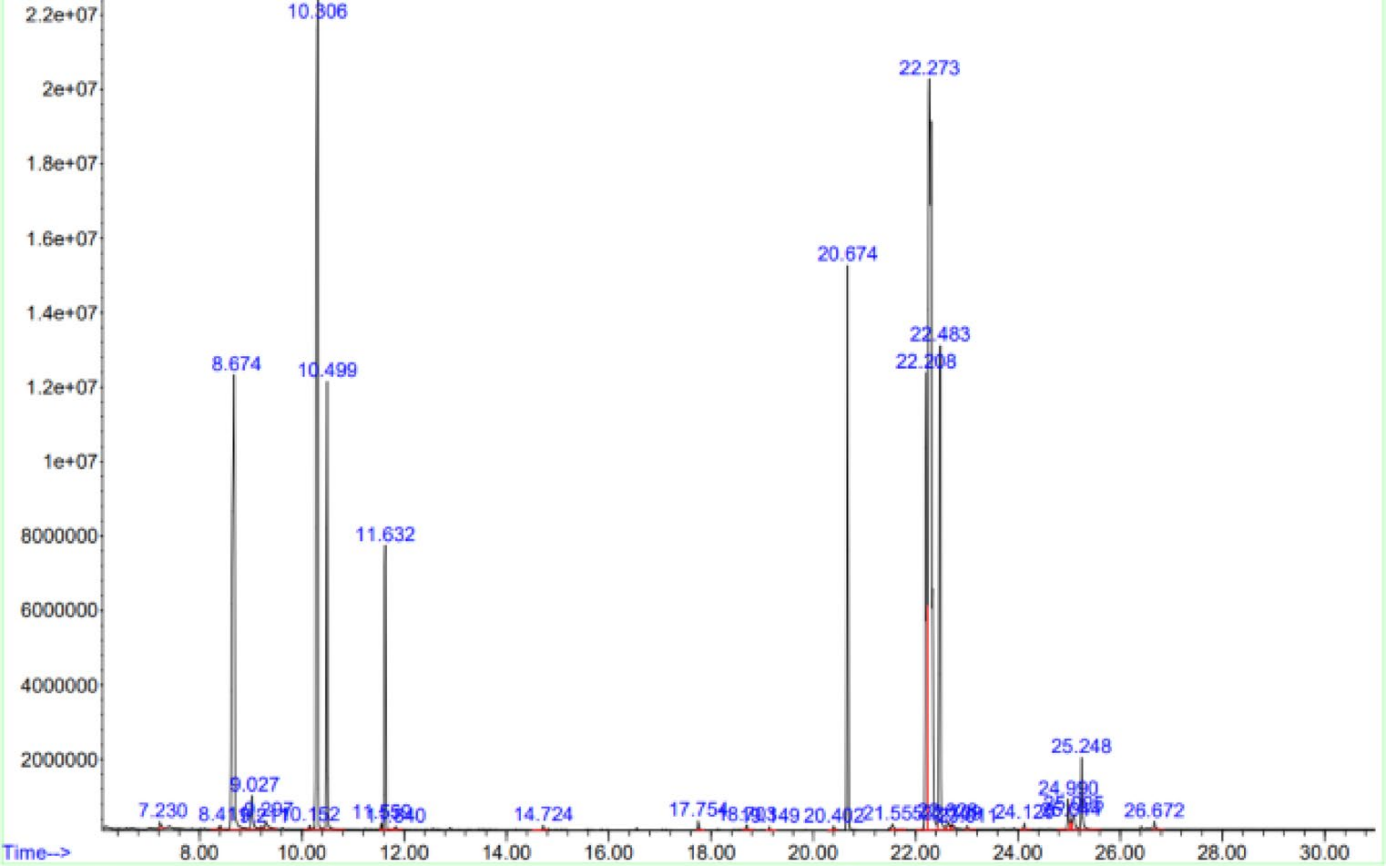
GC–MS mass spectrum analysis of NLC-IB.

The GC–MS chromatogram of the optimized NLC-IB formulation shown in Fig. 7. Comparison with the raw lipid (Fig. 6) enables assessment of fatty acid retention and chemical stability throughout the nanoparticle preparation process.

GC–MS spectra confirmed the presence of stearic, palmitic, and oleic acids in both raw and formulated samples, showing no significant compositional change (p > 0.05). The relative fatty acid composition of raw Illipe butter and the optimized NLC-IB formulation is summarized in Table 3. The major fatty acids identified were stearic and palmitic acids, and their relative proportions were not significantly altered following NLC preparation. The quantitative values reported in Table 3 were directly derived from the integrated peak areas of the GC–MS chromatograms shown in Figs. 6 and 7, ensuring numerical consistency between graphical and tabulated data.

**Table 3:** Fatty acid composition of Illipe butter (IB) and optimized NLC-IB determined by GC–MS.

Fatty acid	Carbon chain	IB (%)	NLC-IB (%)
Palmitic acid	C16:0	18.2	17.9
Stearic acid	C18:0	31.9	31.4
Oleic acid	C18:1	28.6	29.1
Linoleic acid	C18:2	11.4	11.0
Arachidic acid	C20:0	3.2	3.0
Others (< 1% each)	–	6.7	7.6
Total	–	100.0	100.0

Relative fatty acid composition was calculated by normalizing individual GC–MS peak areas to the total peak area of all identified fatty acids in the chromatogram and expressed as percentage composition. This suggests successful encapsulation of Illipe butter lipids without oxidative degradation during processing.^3,4^

### NLC gel formulation properties

Carbopol concentrations (0.5%, 0.75%, 1%) were evaluated for gel base development. The 1% Carbopol base showed superior stability and was used to prepare NLC-IB gel and NLC gel formulation at a concentration of 5% NLC. The E-NLC-IB exhibited pseudoplastic, non-Newtonian behavior with viscosity of 7.47 ± 0.05 Pas and pH 5.65 ± 0.01, appropriate for dermal application.^36^ No significant variations were observed after three freeze–thaw cycles, indicating satisfactory stability.^37^

**Fig. 8:**
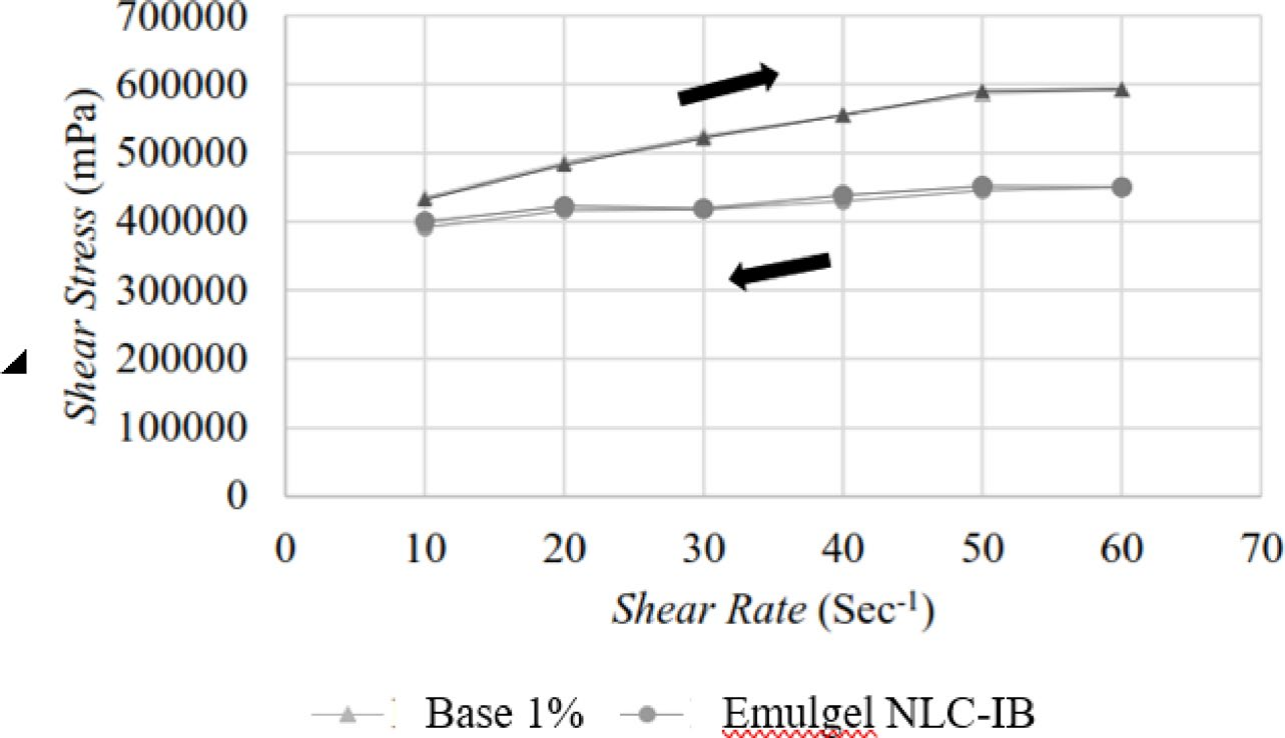
Rheogram of the upper curve ( →) and lower curve ( ←) of shear stress against shear rate.

The rheological behavior of the NLC-IB gel, demonstrating its pseudoplastic flow properties shown in Fig. 8. The upward and downward curves indicate shear-thinning characteristics essential for comfortable topical application.

Both gel and NLC gel formulation demonstrated stable organoleptic characteristics, viscosity, and pH across three freeze–thaw cycles. Rheological analysis confirmed pseudoplastic non-Newtonian behavior, favorable for topical application due to ease of spreading and enhanced residence time.^38^

### Anti-inflammatory activity

**Fig. 9:**
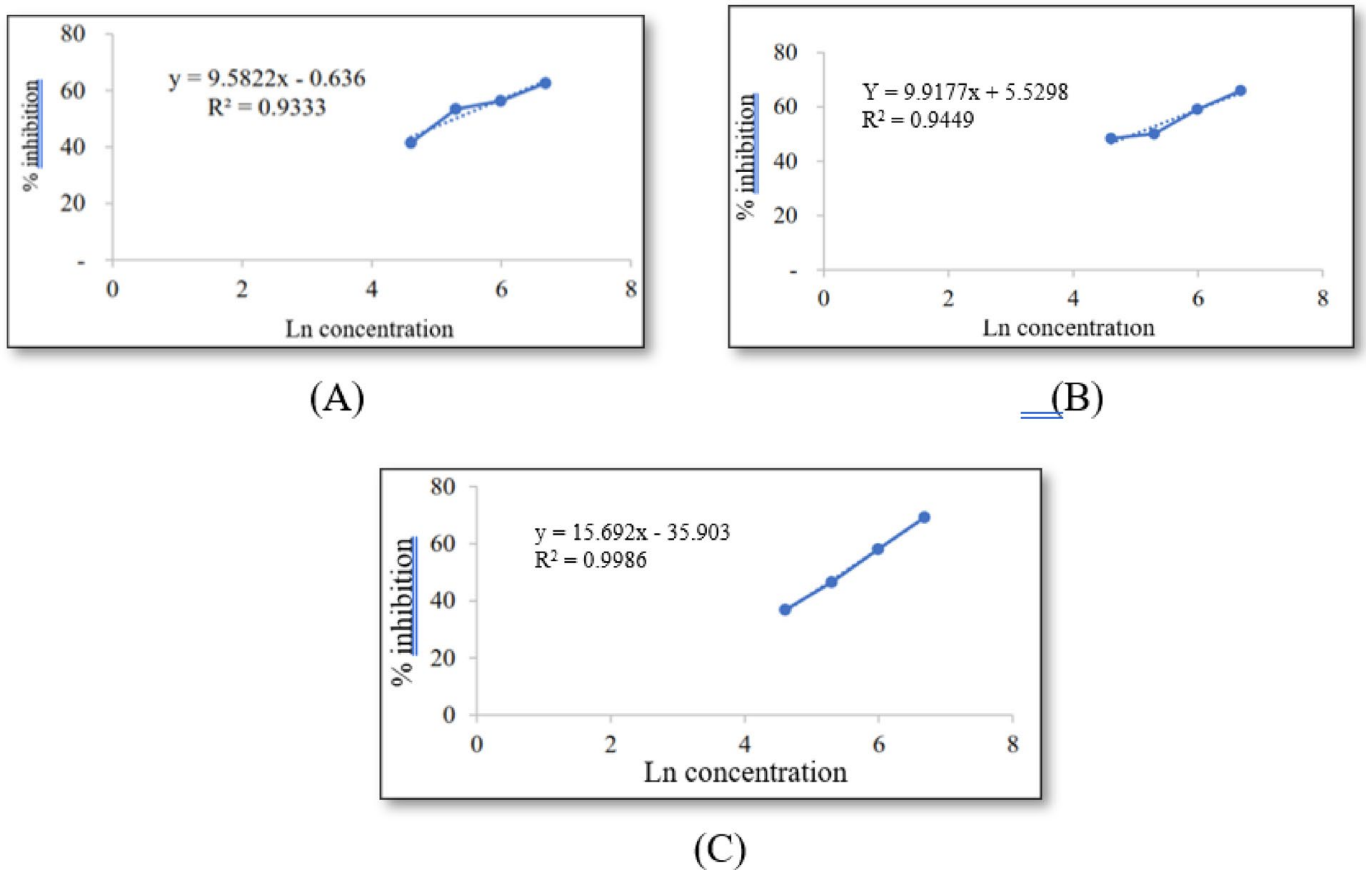
Linear regression graph of anti-inflammatory activity of (**A**) Illipe Butter (IB); (**B**) NLC-IB; (**C**) E-NLC-IB.

Illipe butter (Tengkawang fat), NLC-IB, and E-NLC-IB inhibited > 20% protein denaturation at all tested concentrations. The linear regression curves for the anti-inflammatory activity of Illipe butter, NLC-IB, and E-NLC-IB can be seen in Fig. 9A–C. These graphs provide a comparative assessment of inhibition efficiency and allow determination of IC₅₀ values for each formulation.

The in vitro BSA assay revealed moderate inhibition with IC₅₀ values of 197.23 ppm (Illipe butter), 146.46 ppm (NLC-IB), and 238.49 ppm (E-NLC-IB), indicating improved activity of the nanoformulated system compared to raw lipid. Fatty acids such as stearic and oleic acid have been reported elsewhere to modulate inflammatory pathways; however, the present study does not directly investigate these mechanisms.^39,40^ These findings align with prior studies on lipid-based nanocarriers enhancing anti-inflammatory efficacy.^41^

These results show that Illipe butter can be effectively integrated into nanostructured lipid carriers while maintaining its chemical composition and demonstrating measurable in vitro protein denaturation inhibition.

## Conclusion

This study demonstrates that Illipe butter (*Shorea spp.*) can be incorporated into nanostructured lipid carriers using a Box–Behnken design approach, yielding nanoscale dispersions with preserved fatty acid composition and acceptable physicochemical properties. Nanoformulation modestly improved in vitro protein denaturation inhibition compared with the raw lipid, while incorporation into an NLC gel formulation reduced apparent activity under the tested conditions. Although these findings support the feasibility of using Illipe butter as a natural lipid component in NLC systems, further optimization and biologically relevant evaluations are required to establish its potential for topical anti-inflammatory applications.

The in vitro anti-inflammatory assay indicated that nanoformulation improved the inhibitory effect of Illipe butter compared with the raw lipid, although the activity of the NLC gel formulation was comparatively lower, suggesting that release behavior may be formulation-dependent. Collectively, these findings support the potential use of Illipe butter as a natural lipid component in NLC systems; however, further optimization is necessary to enhance performance in topical dosage forms.

## Limitations of the study

Several limitations should be considered when interpreting the findings of this work such as :

First, although the Box–Behnken Design predicted a particle size of approximately 100 nm, the experimentally obtained size (~ 277 nm) was substantially larger, indicating that critical process variables such as homogenization intensity, sonication amplitude, and lipid polymorphism were not fully captured by the optimization model.

Secondly, the stability evaluation focused solely on short-term freeze–thaw cycles and visual inspection, failing to properly reflect long-term storage, photostability, or humidity impacts pertinent to actual topical products.

Third, the anti-inflammatory activity was assessed exclusively through the bovine serum albumin denaturation assay, offering an indirect and nonspecific measure of anti-inflammatory potential. No cell-based, enzymatic, or live organism models were used to validate biological significance or mode of action.

Fourth, the absence of in vitro release, skin permeation, or retention studies restricts any conclusions about topical bioavailability and therapeutic efficacy. The decreased activity noted for the NLC gel formulation formulation indicates that matrix entrapment or diffusion barriers could affect bioactivity, highlighting the need for additional research via formulation optimization and release kinetics analysis.

Finally, the fatty acid composition of Illipe butter may vary depending on geographic origin, harvesting conditions, and processing methods; therefore, the present findings may not be fully generalizable to all Illipe butter sources.

## Data Availability

Data included in article/supp. material/referenced in article.
